# Surgical Management of Chiari 1.5 in Children: A Truly Different Disease?

**DOI:** 10.3390/jcm13061708

**Published:** 2024-03-15

**Authors:** Ignazio G. Vetrano, Arianna Barbotti, Tommaso Francesco Galbiati, Sabrina Mariani, Alessandra Erbetta, Luisa Chiapparini, Veronica Saletti, Laura G. Valentini

**Affiliations:** 1 Department of Neurosurgery, Fondazione IRCCS Istituto Neurologico Carlo Besta, 20133 Milan, Italy; arianna.barbotti.ab@gmail.com (A.B.); tommaso.galbiati@istituto-besta.it (T.F.G.); sabrina.mariani@istituto-besta.it (S.M.); laura.valentini@istituto-besta.it (L.G.V.); 2 Department of Biomedical Sciences for Health, Università degli Studi di Milano, 20122 Milan, Italy; 3 Department of Neuroradiology, Fondazione IRCCS Istituto Neurologico Carlo Besta, 20133 Milan, Italy; alessandra.erbetta@istituto-besta.it; 4 Department of Diagnostic Radiology and Neuroradiology, Fondazione IRCCS Policlinico San Matteo, 27100 Pavia, Italy; l.chiapparini@smatteo.pv.it; 5 Developmental Neurology Unit, Mariani Foundation Center Foizr Complex Disabilities, Fondazione IRCCS Istituto Neurologico Carlo Besta, 20133 Milan, Italy; veronica.saletti@istituto-besta.it

**Keywords:** Chiari 1.5, Chiari malformation type 1, children, craniovertebral junction, posterior fossa decompression, syringomyelia, tonsils herniation

## Abstract

**Background:** In patients with Chiari 1.5 malformation (CM1.5), a more aggressive disease course and an increased association with craniovertebral junction (CVJ) anomalies has been suggested. The best management of this subgroup of patients is not clearly defined, also due to the lack of specific series elucidating this anomaly’s peculiar characteristics. **Methods:** We evaluated a series of 33 patients (25 females, 8 males; mean age at surgery: 13 years) fulfilling the criteria for Chiari 1.5 diagnosis who underwent posterior fossa decompression and duraplasty (PFDD) between 2006 and 2021. **Results:** Headache was present in all children, five presented central apnea, five had dysphagia, and three had rhinolalia. Syringomyelia was present in 19 (58%) children. Twenty patients (61%) showed various CVJ anomalies, but only one child presented instability requiring arthrodesis. The mean tonsil displacement below the foramen magnum was 19.9 mm (range: 12–30), without significant correlation with the severity of symptoms. Syringomyelia recurred or was unchanged in three patients, and one needed C1–C2 fixation. The headache disappeared in 28 children (84%). Arachnoid opening and tonsil coagulation or resection was necessary for 19 children (58%). **Conclusions:** In our pediatric CM series, the need for tonsil resection or coagulation was higher in CM1.5 children due to a more severe crowding.

## 1. Introduction

The Chiari malformation comprises a heterogeneous group of congenital anomalies [1]. Since its first descriptions [2,3] the spectrum of the disease has expanded and progressively changed. The main characteristic of Chiari malformation 1 (CM1) is the caudal cerebellum ptosis through the foramen magnum, leading to obstruction of cerebrospinal fluid (CSF) outflow. Syringomyelia is associated with CM1 in up to 75% of patients [4]; scoliosis is reported in 50% of cases [5]. Despite often being nonspecific, the clinical presentation of CM1, defined as Chiari syndrome, comprises headaches (mainly triggered by Valsalva maneuvers), ocular and otoneurologic alterations, and ataxia [6]. In the case of bulbar involvement, the most frequent symptoms are gait and balance disorders, limb weakness or dysesthesia, lower cranial nerve disturbances, hiccups, oscillopsia, nystagmus, and central hypoventilation syndrome [7]. Polysomnography is indicated to confirm the diagnosis of sleep apneas at any age, particularly in infants and small children with significant overcrowding of the posterior fossa [8,9]; a strict correlation between radiological findings and polysomnographic results has been suggested as well [10]. Overcrowding is worsened if a craniovertebral junction (CVJ) malformation is associated with CM1. However, it has been recently suggested that the primary CM1 could be secondary to the pre-existence of CVJ instability [11,12], the relationship between CVJ anomalies and CVJ instability is still unclear, and a general agreement on its management, often different from and even independent of CM1, is missing [13,14,15].

More recently, the Chiari 1.5 malformation (CM1.5) has been introduced within a scientific context as a distinct entity [1,16,17]. According to the international consensus document, CM1.5 has been defined as cerebellar tonsils and brainstem herniation below the McRae line, which could also be associated with skeletal deformities of CVJ and the cervical spine (Klippel–Feil anomaly, atlantooccipital fusion, basilar invagination, and retroversion of the odontoid process) [17,18]. Very few studies with a limited number of patients and focusing specifically on CM1.5 are currently available, with a few children sporadically included; meanwhile, the wider pediatric series are reported under the generic CM1 umbrella. Nevertheless, the course of the disease have been reported to be more rapidly progressive in CM1.5 when compared to the classical CM1 counterparts, with affected children more prone to present sleeping disorders [19]. A general impression is that CM1.5 children have earlier symptomatic presentation [20], worse responses to posterior cranial fossa decompression (PCFD), and a higher persistence of syringomyelia [1]. However, this group of patients is still poorly recognized and is treated in the same way as CM1; this is mainly due to the lack of specific literature. With the aim to elucidate the clinical course of CM1.5 children, we present a wide, homogenous series of patients submitted to posterior fossa decompression and duraplasty (PFDD) in a tertiary national referral center. Clinical and radiological findings, postoperative results, and outcomes are discussed in detail, to evaluate the role of associated CVJ alterations.

## 2. Materials and Methods

### 2.1. Patients’ Characteristics

We retrospectively evaluated all patients younger than 18 years operated on at our institution via PFDD for Chiari malformation between 2006 and 2021. Clinical, radiological, and surgical data were retrieved from a prospectively collected database to assess the number of patients with CM1.5. To define the CM syndrome, we collected demographic data and preoperative clinical features (evaluated by the pediatric neurologists) such as headache, brainstem involvement (considered as sleep-disordered breathing and/or oropharyngeal dysfunction), ataxia, and sensorimotor impairment. The preoperative radiological features considered were brainstem downward displacement, syringomyelia extension, platybasia, degree of odontoid retroversion or other CVJ anomalies, and scoliosis. Surgical procedures were also analyzed, along with perioperative complications and the need for second or further surgeries. Finally, the outcome was evaluated according to symptom variations, sleep-disorder and breathing improvement, and syringomyelia (considered as improved, worsened, or stable, according to the last available MRI pictures). All cases in which tonsil descent was secondary to other causes, such as craniosynostoses, hydrocephalus, and brain tumors, were excluded. Children with metabolic tissue disorders were excluded as well.

### 2.2. Instrumental Assessment

The neuroradiological evaluation was based on MRI and CT scans of the craniovertebral junction. All patients underwent at least one preoperative whole-spine MRI to assess the presence and the extension of syringomyelia or to rule out spinal dysraphism, along with brain MRI scans including multiplanar T1-weighted images (w.i.), and T2 w.i. Moreover, a flow-sensitive phase-contrast (PC) technique assessed CSF flow dynamics at the CVJ. We used a CSF flow velocity of 7 cm/s with in-plane velocity encoding in the craniocaudal direction. The first measurement considered the relation of the tonsils with McRae’s line, the virtual line between the basion and opisthion of the foramen magnum. The obex level and the entity of hindbrain displacement were recorded too, to define the Chiari 1.5. In the case of suspected CJV anomalies, a dynamic 3D CT scan was performed to evaluate the clivo-axial angle, the occipital-axial angle, the subaxial cervical lordosis, and the basion-dental and basion-axial interval. We also calculated the posterior fossa volume in selected cases and submitted it to 3DCT to exclude associated craniosynostosis. The function study for instability was performed in all cases using CVJ malformation, mainly via CT with neck flexion or extension, obtained using a gel pad with a head fixation device in the supine position. In a few cases, dynamic MRI was performed to avoid the burden of X-rays. CVJ instability was defined following the Menezes criteria, considering that its definition in children differs from that in adults due to immature supporting structures and ligamentous laxity [21]. In all cases, a non-contrast CT scan was performed before the patient’s discharge to evaluate the extent of decompression and to exclude complications, such as CSF collections or the onset of hydrocephalus. A subsequent clinical and neuroradiological follow-up (brain MRI with PC flow studies and spinal MRI) was indicated at 3 months and 1 year from surgery. Afterward, the follow-up was repeated every 1–3 years, depending on the outcome. In all the cases judged as CM1.5 the following measures were evaluated: odontoid process retroflection (measured as angles), basilar invagination, and caudal displacement of cerebellar tonsils and hindbrain below the foramen magnum.

All very young children (<6 years old) and the ones with suspected obstructive apnea underwent nocturnal polysomnography (PSG). When indicated, the apnea-hypopnea index (AHI), the obstructive apnea index (number of obstructive apneas per hour of sleep time), and the central apnea index (number of central apneas per hour of sleep time) were pre- and postoperatively evaluated. The oxygen saturations and the peak of transcutaneous CO_2_ were measured too.

### 2.3. Surgery and Postoperative Management

All patients underwent a standard preoperative evaluation, including blood tests, and general and neurological clinical assessment. At our center, the surgery was indicated only in children with CM syndrome and/or syringomyelia and/or sleep apneas; in all these cases, a posterior fossa decompression with duraplasty (PFDD) was the treatment of choice. After the suboccipital bone and C1 arches were exposed, the bony decompression was performed with a high-speed drill and bony rongeurs. There was no standard rule regarding the extent of the decompression, which was tailored according to preoperative images to sufficiently enlarge the posterior fossa and to avoid cerebellar sagging. The posterior arch of C1 was always removed. The dura was then incised longitudinally, starting caudally (generally above C2) and proceeding cranially, trying to leave the arachnoid intact. The choice to open it depended on specific intraoperative findings, such as explicit adhesions or CSF flow obstruction visible through the arachnoid, or very low-lying tonsils. Subpial tonsils coagulation was usually reserved for very low tonsillar ectopia (below C2) and/or severe hindbrain dysfunction, since this maneuver could add some morbidity and increase the risk of arachnoid scarring and CSF collections due to arachnoid opening.

### 2.4. Statistical Analysis

Descriptive statistics were reported in terms of absolute numbers and percentages for categorical data and using means with standard deviations (SDs) for continuous data. Associations between variables were investigated with the *t*-test or Pearson’s correlation coefficients with the corresponding *p*-value, as appropriate. *p* values of <0.05 were considered statistically significant and all tests were two-sided. STATA statistical software, version 16 (StataCorp. 2019. Stata Statistical Software: Release 16. StataCorp LLC, College Station, TX, USA) was used for the statistical analysis.

## 3. Results

Among 147 children with a diagnosis of Chiari malformation submitted to surgery during the 15 years analyzed, 33 fully satisfied the inclusion criteria and were therefore considered as Chiari 1.5. There were 25 females and 8 males (ratio 3:1), with a median age at surgery of 13 years (range: 5–18). The medium follow-up was 30 months (range: 9–124 months). Demographic and clinical data are presented in Table 1.

Regarding this specific subgroup of patients, the first symptom leading to diagnosis was the headache, which was usually not responsive to common painkillers; meanwhile, only 23 patients out of 33 showed a typical headache after Valsalva maneuvers. Eight patients were submitted to PSG, which confirmed central apnea in only five of them. Five children presented dysphagia and three presented rhinolalia. Seven patients showed preoperative scoliosis (21%). Syringomyelia was present in 19 (58%) patients: holocord in nine and limited to the cervicothoracic spine in five. The remaining five children showed only cervical syringomyelia; interestingly, the group of patients with syringomyelia showed relatively higher cerebellar tonsil descent (mean: 16.68 mm) than the group without syrinx (mean: 20.7 mm) (*p* = 0.017). The degree of tonsil descent did not correlate with any of the other variables analyzed. Hindbrain displacement was not statistically correlated to any of the other variables.

In all children, the hindbrain was downward displaced for more than 1/3 of its length, with the obex under the foramen magnum (mean displacement: 15.16 mm). Regarding CVJ anomalies, 20 out of 33 CM1.5 children (61%) presented different kinds of alterations: 17 with odontoid retroflection, 1 with os odontoideum, 1 with cleft of the posterior arch of C1, and 1 with basilar invagination. Nevertheless, only one girl presented signs of CVJ instability. The analysis evidenced a significant correlation between the odontoid process retroflection angle and the presence of sleep apneas. In contrast, odontoid process retroversion angles and the degree of basilar invagination seem to be correlated with the presence of pre-operative syringomyelia (*p* < 0.05).

**Figure 1:**
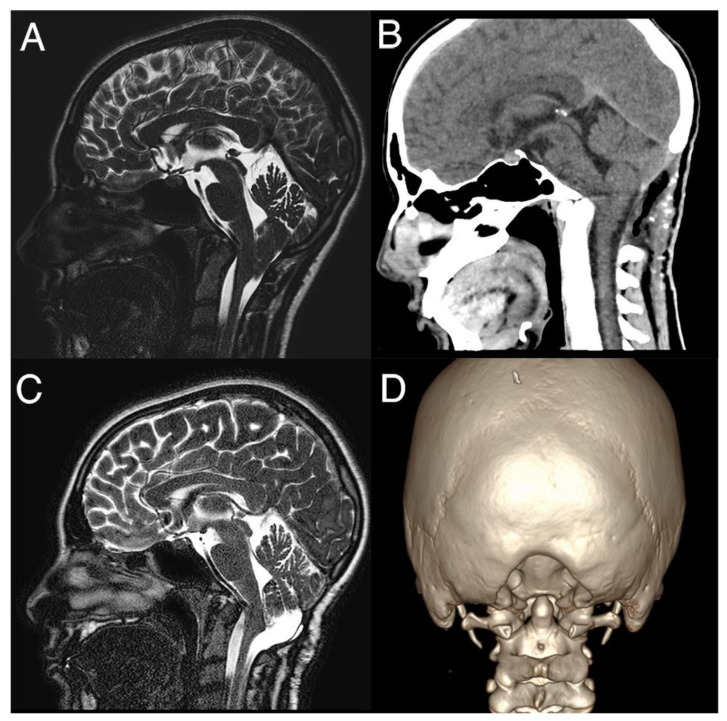
Chiari 1.5 malformation: the preoperative T2-weighted mid-sagittal scan demonstrates the displacement of the cerebellar tonsils and hindbrain below the foramen magnum (**A**). The patient was submitted to PFDD: the postoperative CT scan (**B**) with 3D reconstruction (coronal view in (**D**)) showed the entity of bony decompression, with CSF flow in the posterior fossa and reduction of tonsil descent (**C**).

All children underwent PFDD and C1 laminectomy (Figure 1).

Tonsil coagulation was performed in 12 patients and subpial resection in another 7. After assessing cerebellar pulsations as a sign of restored CSF flow, the duraplasty was performed in all cases using allograft patches, mainly bovine or equine pericardium, with non-resorbable stitches. We did not experience major neurological morbidities or mortality after surgery. One child presented a local wound infection, treated with antibiotic therapy only. Two children presented CSF leak, which was conservatively managed in one case (only wound suture) and required a second surgery in another.

### Outcome after Surgery

The global outcome of surgery was favorable in both groups (Table 2). In 28 children (84%), headache disappeared after the surgical treatment, and 4 of them experienced a remarkable improvement. Only one patient failed to improve in terms of bulbar symptoms and headache after surgery, with persistent obstructive apnea, dysphagia, and rhinolalia. Bulbar symptoms disappeared at long-term follow-up in all children who had them at presentation, except one. Scoliosis improved in five out of seven children affected; in the remaining two it remained stable but needed surgical correction for the severe degree already present before surgery. Syringomyelia markedly improved or fully disappeared in 16 out of 19 children, while it recurred in two cases and remained unchanged in one. Three patients were submitted to a second surgery to enlarge bony decompression due to persistent bulbar symptoms (one child), headache, and/or syrinx worsening at the 1-year follow-up.

**Figure 2:**
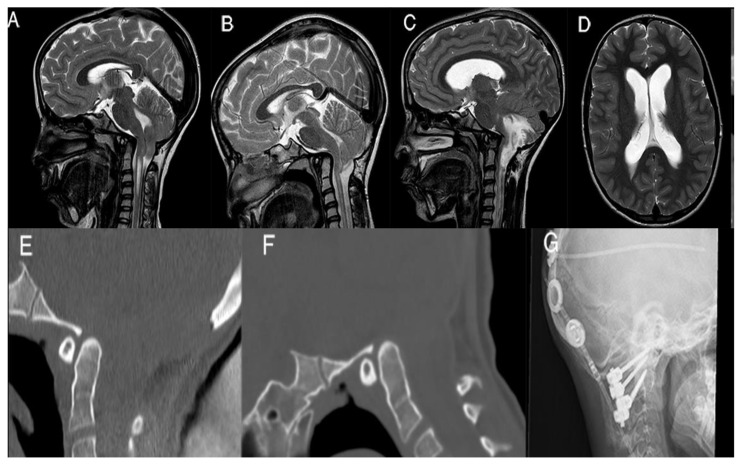
An 11-year-old girl with severe tonsil descent, hindbrain herniation, and cervical syringomyelia ((**A**), preoperative T2-w.i. sagittal MRI). The first MRI ((**B**), T2-w.i. sagittal scan) depicted flow restoration in the posterior fossa and syrinx reduction. However, six months after surgery, the child experienced the onset of acute headache and neck pain. A new MRI scan showed tonsil infarct, cerebellar swelling, and hydrocephalus ((**C**), T2-w.i. sagittal scan). The dynamic cervical CT scan (**D**,**E**) demonstrated slight instability. The patient underwent ventriculoperitoneal shunt positioning with a programmable valve and C1–C2 fixation ((**F**), as shows the postoperative X-ray lateral plan (**G**)).

In Figure 2 we present one of these failures, the case of an 11-year-old girl, who experienced the acute onset of hydrocephalus 3 years after PFDD, secondary to subacute tonsillar ischemia; urgent scans also demonstrated worsening of the basilar invagination. CVJ instability was demonstrated with dynamic CT scans. Therefore, she underwent a ventriculoperitoneal shunt and C1–C2 posterior arthrodesis, with clinical and neuroradiological improvement at 1-year follow-up. Nevertheless, her preoperative scoliosis remained unchanged after atlantoaxial fixation and needed surgical correction (Figure 2).

## 4. Discussion

Our retrospective study shows that CM1.5 children could be assimilated into the CM1 patients in terms of response to PCFD, despite a more severe neuroradiological presentation. We have found no specific signs or symptoms peculiar to the Chiari 1.5 malformation. However, to date, no large series are focusing specifically on CM1.5, especially in children. Since the first descriptions of this subset of CM1 patients [17], CM1.5 has been now accepted as a separate entity, also according to the recent consensus [18]. Our first impression is that children with CM1.5 usually present a more severe degree of headache and a slight increase in severe symptoms, such as the bulbar ones. In our experience, CM1.5 children have a higher risk of becoming symptomatic along follow-up and consequently receiving a surgical indication (data presented, not already published). This attitude has also been reported by Tubbs et al. [17], whereas we have not found a significant statistical correlation between the degree of tonsils or hindbrain displacement and the severity of the clinical presentation. A more significant difference is related to the need for tonsil coagulation or resection: in our series, 19 children out of 33 (58%) CM1.5 received coagulation of the cerebellar tonsils. Marzerat et al., in a multicentric French cohort study of 255 CM1 children published in 2022, showed a rate of tonsillectomy of 13.7% [22], significantly lower than the rate we present here. This may be explained by the fact that, in the current series, the mean tonsil displacement below the McRae’s line was 19.9 mm, and considering also the hindbrain herniation, these maneuvers have been dictated by the increasing difficulty of restoring a valid CSF flow across the very severely crowded foramen magnum. In our cohort, the rate of syringomyelia persistence or recurrence was 18.5%, similar to the 13.6% recurrence rate in the Tubbs CM1–CM1.5 series, and higher than the reported one for CM1 children (6%). It is worth noting that in our institution we try to restrict this maneuver to very selected cases with severe crowding and very low-lying tonsils, because we agree that intra-arachnoid manipulation and tonsillectomy may increase the risk of future arachnoid scars, which reduces the CSF flow and causes the late recurrence of syringomyelia, as highlighted by a panel of experts in Chiari malformation management [15].

Another point of interest regarding CM1.5 children is the exact incidence of bulbar symptoms, mainly regarding central apnea. Losurdo et al. showed, in a general series of CM1 patients, that the prevalence of sleep-disordered breathing was 24%, slightly lower than that reported in the literature [8]. When focusing on CM1.5 children, Sader and coauthors evidenced that 87.5% of patients experienced preoperative snoring or witnessed apnea, which significantly improved after posterior cranial fossa decompression [19]. However, these findings were based on a limited series of seven children. In our series, eight children performed preoperative PSG, which confirmed central apnea in 5 cases out of 33 (15%). Among them, only in one child did apnea persist after surgery (PSG). Interestingly, as reported, higher degrees of odontoid process retroflexion are associated with the presence of sleep apneas; according to this, when a radiological picture of CM1.5 is seen, a PSG should be ordered. Moreover, PSG was usually not considered a routine examination in the past due to the lack of specific guidelines and the need for compliance from the patients. Therefore, the prevalence of central apnea in our series could be underestimated.

In the presence of bulbar signs and symptoms, some authors invoke the role of ventral compression and CVJ anomalies [11,12], which are sometimes associated with CM1 in general, especially in CM1.5 with associated CVJ malformation. In our series, 20 out of 33 children (61%) presented some degree of CVJ alteration, mainly odontoid retroflection or retroversion, which are correlated retrospectively with the presence of sleep apneas or pre-operative syringomyelia. The odontoid process retroversion, present in 17 children among the 33 (51%) did not correlate with the degree of tonsillar or brainstem herniation; furthermore, other groups reported the lack of a similar correlation [17]. Despite the higher prevalence of CVJ malformations observed in CM1.5 (61%), true CVI in-stability was identified in only 1 girl among all 20 children with associated CVJ anomalies. In this unique case, the CGH array analysis revealed chromosomal rearrangements: a deletion of approximately 3.8 Mb at the 14q32.32-q32.33 level, which likely contributed to articular laxity, and a duplication of about 923 Kb at the 22q13.33 level. In this girl, PFDD initially led to a 3-year improvement; however, there was subsequent progressive deterioration. This deterioration resulted in cerebellar ischemia and hydrocephalus requiring the placement of a shunt, and ultimately C1-C2 posterior fixation. This case emphasizes the importance of precise evaluation and diagnosis when dealing with suspected CVJ instability. Recently, the consensus document produced by a panel of experts confirmed the complexity of the management of CVJ in CM patients and the need to look for CVJ instability in selected patients [15]. In this context, the high anatomical degree of variation amongsts children, according to age, may impact the preoperative assessment. Different criteria have been proposed for CVJ instability related to the atlantoaxial instability with an atlantodental interval exceeding 5 mm (mainly in children younger than 8 years) or the occipitoatlantal instability and separation of the lateral masses of the atlas by more than 7 mm, along with abnormal CVJ dynamics [21,23]. Some studies suggested that basilar invagination is a predictor of the need for occipitocervical fusion surgery [24]. During the last few years, Goel et al. proposed a new pathogenic mechanism in which the Chiari malformation is secondary to constant association with atlantoaxial joint instability (even if not radiological evident, or in the absence of clinical manifestation), thus advocating the necessity of performing C1-C2 fixation without posterior fossa decompression [11,12,25]. Atlantoaxial instability with subluxation has been reported in up to 38% of CM1 children [13], and it is related to an increased incidence of long-tract impairment [26]. Our series does not reflect such findings, considering that we had very good outcomes with PFDD and did not experience the need for craniovertebral fixation in any of the 147 cases, excluding the abovementioned case. Nevertheless, we believe that the careful preservation of the C2 muscular and ligamentous complex is mandatory to reduce the risk of instability. A considerable agreement was achieved by the consensus on Chiari and syringomyelia in 2019, to reserve surgical indication only to symptomatic children with basilar invagination/impression basilaris-related CM 1.5, excluding CVJ fixation in CM1 children without documented CVJ instability or hypermobility [15].

### Limitations

The main limitation of the current study is represented by its retrospective nature and the lack of a clear definition of the exact radiological parameters to define Chiari 1.5. Nevertheless, we analyzed a monocentric and homogeneous series, with the vast majority of procedures performed by the senior surgeon. Considering that only some children executed PSG, this may have underestimated the prevalence of central apnea, which is not always clinically evident.

## 5. Conclusions

Chiari 1.5 comprises a particular subset of CM1 patients; however, there are no peculiar symptoms and signs, and affected children are slightly more prone to develop severe symptoms, such as bulbar signs. In our large series, we experienced a higher need to perform tonsil resection or coagulation to gain space and a slightly higher need to redo surgery in CM1.5. On the other hand, CM1.5 children, despite the higher incidence of associated CVJ malformations, did not present an increased CVJ instability. Nevertheless, as discussed before, odontoid process retroversion or retroflection should raise the suspicion of associated sleep apneas and syringomyelia, which should be investigated accordingly. Further studies using the same precise neuroradiological definition applied here are necessary to prospectively evaluate whether CM1.5 represents a different clinical and surgical entity than the classical CM1, which is more prone to become symptomatic.

## Figures and Tables

**Table 1:** Demographic and clinical details of CM 1.5 compared to CM1 children.

	Chiari 1	Chiari 1.5
	n. 114	n. 33
Mean age (years)	10.3	13.2
Sex (male/female)	56/58	8/25
Headache	52%	61%
Ataxia	20%	16%
Motor deficits	17%	10%
Hypo/Paresthesia	15%	19%
Bulbar signs	7%	16%
Scoliosis	16%	19%
Syringomyelia	58%	61%

**Table 2:** Outcome comparison among CM1 and CM1.5 children.

	Chiari 1	Chiari 1.5
	n. 114	n. 33
Mean follow-up (months)	32	30
CVJ malformations CVJ instability, fixation: 0.7%	10% -	61% 1/11
Mean tonsil descent	12.3 mm	19.9 mm
Bulbar signs Improvement	7% 100%	16% 97%
Scoliosis Improvement	16% 84%	19% 71%
Syringomyelia	58%	61%
Improvement Failure, reoperation	79% 6%	84% 16%

## Data Availability

Data supporting reported results can be found in publicly archived datasets generated during the study at the Fondazione IRCCS Istituto Neurologico Carlo Besta (https://zenodo.org/communities/besta/?page=1&size=20, accessed on 1 March 2024).
